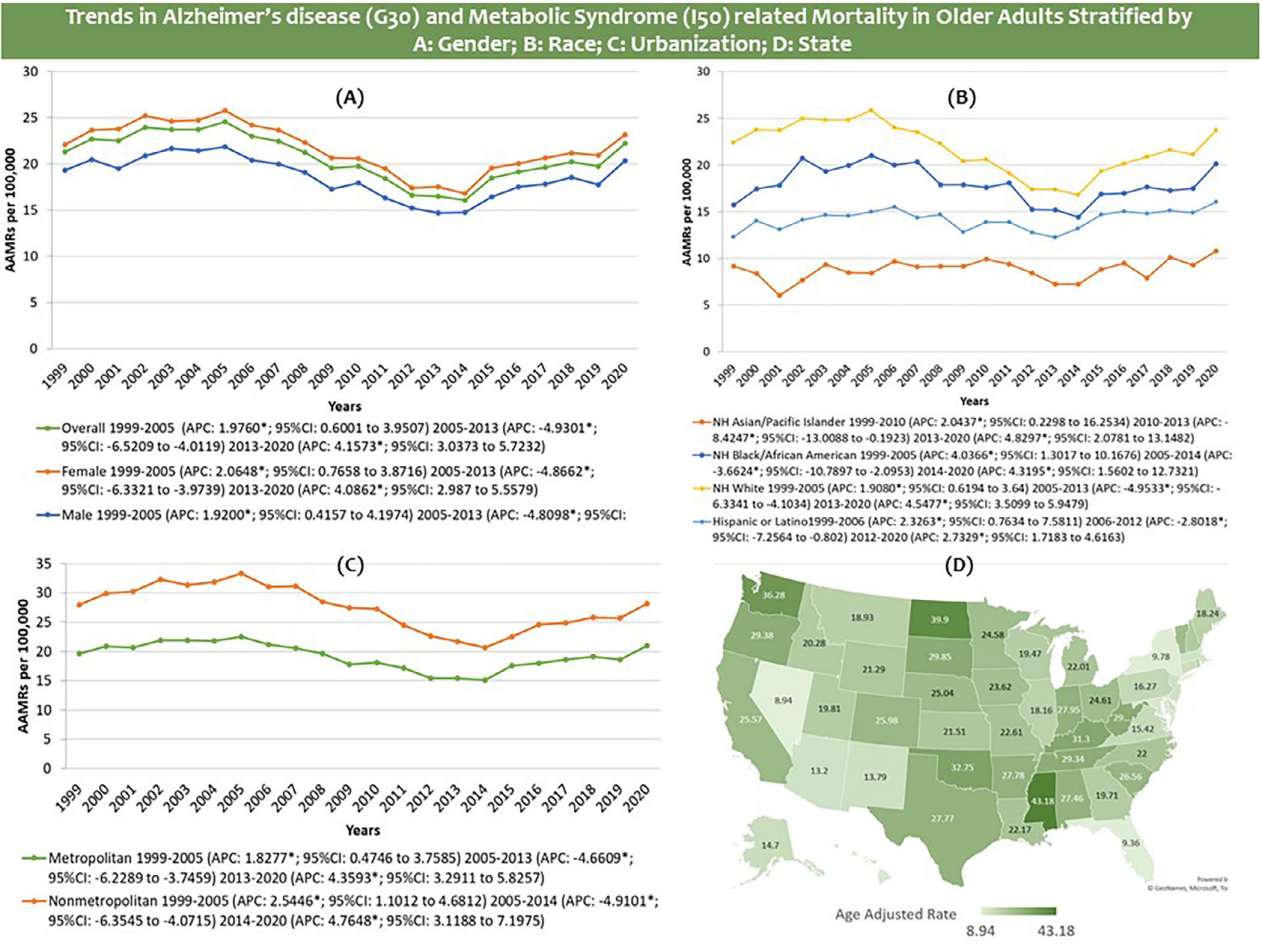# Trends in Alzheimer's disease and heart failure‐related mortality among older American adults: Insights from the CDC WONDER database

**DOI:** 10.1002/alz70860_102012

**Published:** 2025-12-23

**Authors:** Abdul Moeed, Muhammad Ahmed Ali Fahim, Syeda Farwa Zaidi, Farah Yasmin

**Affiliations:** ^1^ Dow Medical College, Dow University of Health Sciences, Karachi, Karachi, Sindh, Pakistan; ^2^ Yale School of Medicine, New Haven, CT, USA

## Abstract

**Background:**

Alzheimer's disease is one of the leading causes of death among the elderly in the United States with heart failure sharing similar risk factors. This study investigated trends and disparities in Alzheimer's disease mortality among older adults with heart failure from 1999‐2020 in the United States.

**Method:**

Using ICD‐10 codes death certificate data from the Centers for Disease Control and Prevention Wide‐Ranging OnLine Data for Epidemiologic Research database was retrieved for patients aged ≥ 65 years between 1999‐2020. Age‐adjusted mortality rates (AAMRs), per 100,000 people, and Annual Percentage Change (APCs) with their respective 95% Confidence Intervals (CI) were also calculated. Data was stratified by year, gender, race and geographical distribution.

**Result:**

Alzheimer's disease with coexisting heart failure was responsible for 192,459 deaths between 1999‐2020. Overall the AAMR increased from 21.32 in 1999 to 24.56 in 2005 (APC: 1.9760*; 95% CI: 0.6001 to 3.9507) after which a significant decrease to 16.52 by 2013 was observed (APC: ‐4.9301*; 95% CI: ‐6.5209 to ‐4.0119). AAMRs decreased from this point forward reaching 22.21 in 2020 (APC: 4.1573*; 95% CI: 3.0373 to 5.7232). Women had higher AAMRs than men (21.57 vs 18.41). Among racial groups, the Non‐Hispanic (NH) White (21.62) population had the highest AAMRs followed by NH Black/African American (17.87), Hispanic/Latino (14.3) and NH Asian/Pacific Islander (8.96). Furthermore, AAMRs also varied by census region (West: 24.05; Midwest: 22.83; South: 21.1; Northeast: 13.38). Moreover, nonmetropolitan areas had higher AAMRs than metropolitan areas (27.23 vs 19.09). States in the top 90th percentile such as Kentucky, Oklahoma, Washington, North Dakota and Mississippi had AAMRs that were three times higher relative to states in the lower 10th percentile including Nevada, Florida, New York, District of Columbia and Hawaii.

**Conclusion:**

Alzheimer's disease mortality with associated heart failure has shown considerable variation in adults ≥ 65 years. AAMRs were highest in women, NH Whites, residents of the West and nonmetropolitan patient populations. Targeted interventions and a more holistic approach to patient management are essential in achieving favorable outcomes for vulnerable groups.